# Nogo-B regulates migration and contraction of airway smooth muscle cells by decreasing ARPC 2/3 and increasing MYL-9 expression

**DOI:** 10.1186/1465-9921-12-14

**Published:** 2011-01-21

**Authors:** Wujian Xu, Weijun Hong, Yan Shao, Yunye Ning, Zailong Cai, Qiang Li

**Affiliations:** 1 Department of Respiratory Diseases, ChangHai Hospital, Second Military Medical University, Shanghai 200433, China; 2 Clinical Research Center, ChangHai Hospital, Second Military Medical University, Shanghai 200433, China

## Abstract

**Background:**

Abnormal proliferation, apoptosis, migration and contraction of airway smooth muscle (ASM) cells in airway remodeling in asthma are basically excessive repair responses to a network of inflammatory mediators such as PDGF, but the mechanisms of such responses remain unclear. Nogo-B, a member of the reticulum family 4(RTN4), is known to play a key role in arteriogenesis and tissue repair. Further studies are needed to elucidate the role of Nogo-B in airway smooth muscle abnormalities.

**Methods:**

A mouse model of chronic asthma was established by repeated OVA inhalation and subjected to Nogo-B expression analysis using immunohistochemistry and Western Blotting. Then, primary human bronchial smooth muscle cells (HBSMCs) were cultured *in vitro *and a siRNA interference was performed to knockdown the expression of Nogo-B in the cells. The effects of Nogo-B inhibition on PDGF-induced HBSMCs proliferation, migration and contraction were evaluated. Finally, a proteomic analysis was conducted to unveil the underlying mechanisms responsible for the function of Nogo-B.

**Results:**

Total Nogo-B expression was approximately 3.08-fold lower in chronic asthmatic mice compared to naïve mice, which was obvious in the smooth muscle layer of the airways. Interference of Nogo-B expression by siRNA resulted nearly 96% reduction in mRNA in cultured HBSMCs. In addition, knockdown of Nogo-B using specific siRNA significantly decreased PDGF-induced migration of HBSMCs by 2.3-fold, and increased the cellular contraction by 16% compared to negative controls, but had limited effects on PDGF-induced proliferation. Furthermore, using proteomic analysis, we demonstrate that the expression of actin related protein 2/3 complex subunit 5 (ARPC 2/3) decreased and, myosin regulatory light chain 9 isoform a (MYL-9) increased after Nogo-B knockdown.

**Conclusions:**

These data define a novel role for Nogo-B in airway remodeling in chronic asthma. Endogenous Nogo-B, which may exert its effects through ARPC 2/3 and MYL-9, is necessary for the migration and contraction of airway smooth muscle cells.

## Background

Airway remodeling in chronic asthma is characterized by epithelial detachment, subepithelial fibrosis, mucus hyperplasia, angiogenesis, airway edema, changes in the cartilage, and most obviously, an increase in airway smooth muscle mass. It is believed that abnormalities in proliferation, apoptosis, migration, secretion, and contraction of smooth muscle cells (SMCs) all play roles in airway smooth muscle remodeling, and contribute to airway hyperresponsiveness [1,2].

The cause for such abnormalities is complex and depends on a network of inflammatory mediators and cytokines. The levels of some mediators, such as PDGF and TGF-β, are greatly elevated in the lung of asthmatic patient and are thought to play important roles in airway smooth muscle remodeling [3,4]. In vitro studies have shown that PDGF is a potent SMC mitogen that can promote proliferation and migration while switching cells to an "immature" phenotype and, therefore, decreasing the contractility of the cells. However, the precise mechanisms underlying these processes remain unclear [5,6].

Reticulons (RTNs) are a family of proteins that include four family members, RTN 1, 2, 3, and 4. In mammals, the RTNs are mainly localized to the endoplasmic reticulum (ER) and are involved in tubulogenesis of the ER and membrane curvature [7,8]. Different isoforms of the RTN family have distinct functions. Recently, the RTN 4 isoforms, also called Nogo, have been demonstrated to be vital mediators of a variety of cellular responses and tissue repair. The RTN 4 family is expressed in three splice variants including Nogo-A, -B, and -C. Nogo-A is primarily expressed in the central nervous system and is identified as a potent inhibitor of axonal growth and repair [9]. Nogo-C exists mainly in skeletal muscle, whereas Nogo-B is widely expressed in peripheral tissues including those of lung and vascular systems [10]. Mice deficient in Nogo-B exhibited an exaggerated neointimal proliferation that could be rescued by adenoviral-mediated gene transfer of Nogo-B [11]. In addition, Nogo-B is necessary for modulating macrophage infiltration and expressing inflammatory mediators macrophage infiltrating and inflammatory mediators' expression for tissue repair after ischemic injury. All of these factors observations indicate that Nogo-B plays a pivotal role in vascular remodeling and tissue repair [12]. Airway smooth muscle remodeling in asthma is basically a SMC repair response to inflammatory mediates and cytokines, the role of Nogo-B in the process of airway smooth muscle remodeling has not yet been reported.

We evaluated the role of Nogo-B in ASM in a mouse model of chronic asthma and then determined the effects of Nogo-B on PDGF-induced proliferation, migration and contraction of HBSMCs *in vitro *using a siRNA strategy. Proteomic analysis was then performed to unveil the underlying mechanisms. Our results demonstrate a novel mechanism through which Nogo-B regulates airway smooth muscle cells.

## Materials and methods

### Animal models

Four to six-week-old male BALB/c mice (Shanghai Laboratory Animal Company, Shanghai, China) were used in our experiments. The mice were sensitized intraperitoneally with Ovalbumin (OVA, Sigma Aldrich) in alum (Days 0, 7, and 14). Control mice received the same volume of PBS in alum, as previously described [13]. Chronic allergic airway remodeling was induced when mice were subsequently exposed to aerosolized OVA challenges three times a week from Days 21 to 72. Mice were sacrificed at the indicated times and the lungs were harvested, either into 4% formalin for histological evaluation or snap-frozen into liquid nitrogen for protein preparations. Animals were treated humanely according to Institutional Animal Care procedures.

### Cell culture

Primary human bronchial smooth muscle cells (HBSMCs) and smooth muscle growth medium (SmGM) were purchased from ScienCell (U.S.A). HBSMCs were cultured in SmGM containing 5% FBS. The cells were incubated at 37°C in a 5% CO_2_-humidified atmosphere. Cells from passages 4 to 10 were used for the experiment. PDGF-BB was purchased from R&D (U.S.A) and dissolved in PBS to yield a stock solution of 1 μg/ml.

### Histological examination

Mouse lung tissues were collected and embedded in paraffin for histological analysis. Lung sections were stained with hemotoxylin and eosin (H-E) for examination of airway remodeling. For the immunohistochemistry, 5 μm thick sections were cut, and the Envision method was performed according to the instructions (DakoCytomation, Denmark). Anti-SM-22 antibody, anti-Nogo-B antibody were applied. 3, 3'-Diaminobenzidine was used as a chromogen with a subsequent hematoxylin counter-stain.

### RNA interference

For Nogo-B knockdown, Nogo-B siRNA1 (NOGOi-1 forward: AGA AAU UAU UAA UUA CAA A, reverse: UUU GUA AUU AAU AAU UUC U) and Nogo-B siRNA2 (NOGOi-2 forward: GAU CGU UGU UAG AUC UUU A, reverse: UAA AGA UCU AAC AAC GAU C) were employed. The control siRNA in experiments refers to a negative control siRNA (NEGi forward: UUC UCC GAA CGU GUC ACG U, reverse: ACG UGA CAC GUU CGG AGA A). All of the above siRNAs were designed and synthesized by Qiagen (Germany). For 6-well plate transfection, human bronchial smooth muscle cells were transfected with 300 ng siRNA using 12 μl Hiperfect (Qiagen) according to the manufacturers' instructions. Efficacy of siRNA interference of Nogo-B was assayed at 24 to -60 h post-transfection by Western blotting.

### Western blotting analysis

The protein concentration was determined using the Bio-Rad protein assay system. HBSMCs were dissolved and boiled in Laemmli buffer for 5 min. Twenty micrograms of proteins were subjected to electrophoresis in 12% SDS-PAGE, transferred to nitrocellulose membrane, blocked in PBS containing 5% skimmed milk for 2 h at room temperature and then reacted with the primary antibody of Nogo-B, ARPC 2/3 (Abcam, Britain) or MYL-9 (Santa Cruz, U.S.A). The quantity of expressed protein was normalized to GAPDH.

### Real-time PCR

Real-time PCR analysis was performed using the LightCycler^® ^2.0 Real-Time PCR system (Roche, Switzerland) and the SYBR PrimeScriptTM RT Reagent Kit (TaKaRa, Japan) following the manufactures' instructions. The primers and reaction conditions were shown as below: Nogo-B, forward (5'-GCA GGG GCT CCG GCT CAG TG-3') and reverse (5'-GTT CAC ATG ACC AAG AGC CAG-3');β-actin, forward (5'-GGA CTT CGA GCA AGA GAT GG-3') and reverse (5'-AGC ACT GTG TTG GCG TAC AG-3'), 40 cycles. The Nogo-B mRNA expression was normalized by the housekeeping gene β-actin.

### Proliferation assay

Cell proliferation assays were performed using Cell CountingKit-8 (Dojindo, Japan). Cells were plated in 96-well plates at 3.5×10^3 ^cells per well and cultured in growth medium with 2% FBS. At the indicated time points (24 h, and 48 h,), the cell numbers in triplicate wells were measured as the absorbance at 450 nm from WST-8(2-(2-methoxy-4-nitrophenyl)-3-(4-nitrophenyl)-5-(2,4-disulfophenyl)-2H-tetrazolium, monosodium salt)

### Boyden chamber migration

Cell migration assays were performed using Millicell cell culture inserts (Millipore, U.S.A) [14]. HBSMCs, which had been treated with siRNA for 48 h, were serum-starved overnight. PDGF-BB (20 ng/ml) and 10% FBS were prepared in SmGM and added to the bottom chambers. HBSMCs (5×10^4^) in serum free SmGM were added to the upper chambers. After 5 h of incubation at 37°C, cells on both sides of the membrane were fixed and stained with 0.1% crystal violet. Cells on the upper side of the membrane were removed with a cotton swab. The average number of cells per field was determined by counting the number of cells in four high-power (×200) fields from the lower side of the membrane.

### Gel contraction assay

The contractility of the cultured HBSMCs was examined using a gel contraction assay [15]. For each 6-well plate, collagen solution was prepared by mixing 450 μl of ice-cold type I collagen (5 mg/ml, Shengyou biotechnology, China) with 53 μl 10× PBS, pH was adjusted to 7.4 with 0.1 M NaOH. HBSMCs pretreated with siRNA for 48 h were seeded at a density of 3×10^5 ^cells/ml; 1.5 ml of gel suspension was poured into a 6-well culture pate. The gels were cultured in 2 ml of 5% FBS SmGM overnight added with PDGF or PBS and then started the contraction assay. Gel surface images were captured with a digital camera 24 h later. Contraction of the gel was then evaluated by measuring its surface area with Image-Pro Plus 6.0 (Media Cybernetics, Inc. U.S.A). Data were expressed as percentage of the original gel size.

### Proteomic analysis

Proteomic analysis was performed, as previously described [16]. Briefly, HBSMCs transfected with NEGi or NOGOi-2 from three 60 mm cell culture dishes were, respectively, pooled as one sample. Total proteins of the cell samples were homogenized and treated with 2-D Clean-Up Kit (Amersham), following the manufacturer's protocol. Protein (600 μg) from each sample was loaded into DryStripTM (13 cm, pH 3-10; Amersham) and isoelectric focusing was performed on MultiphorTMII (Amersham) at 18°C. Two 15-min equilibration steps were carried out using equilibration tubes. After equilibration, the strips were transferred onto 15% polyacrylamide gels for second dimensional (2D) SDS-PAGE. The 2ndD gels were silver stained and digitized using an imaging system - ChemiImagerTM 5500 (Alpha Innotech, San Leandro, USA). Image analysis was conducted using the ImageMasterTM 5.0 (Amersham). Only significantly different spots (three-fold increase or decrease) were selected for analysis by mass spectrometry. Target proteins were excised and digested. Peptides were then extracted, dried and subjected to MALDI-TOF MS analysis. Data from MALDI-TOFMS MS were analyzed using MASCOT (Matrix Science, London) search software. The following parameters were used in the search: mammalian, protein molecular mass ranged from 700 to 32, 000Da, trypsin digest with one missing cleavage, peptide tolerance of 0.2, MS/MS tolerance of 0.6 Da and possible oxidation of methionine.

### Statistical analysis

All values were expressed as the mean ± SD of n observations (n > = 3).Statistical analyses between groups were performed using one-way analysis of variance or Student *t *tests between two groups, as appropriate. P < 0.05 was considered statistically significant.

## Results

### Down-regulation of Nogo-B in airway smooth muscle of chronic asthmatic mice

**Figure 1:**
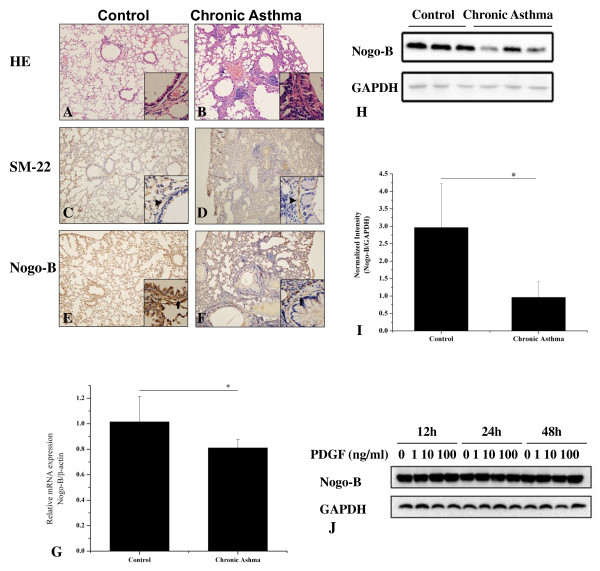
**Down-regulation of Nogo-B in airway smooth muscle of chronic asthmatic mice**. Repeated sensitization and provocation resulted in airway remodeling (**B**) compared to wild type littermates (**A**). Immunohistochemistry analysis demonstrated increased expression of SM-22 in chronic asthmatic mice (**D**) relative to wild type littermates(**C**). Arrow head indicated SM-22 expression in the smooth muscle layer of the airways. Nogo-B expression in control (**E**) and chronic asthmatic mice (**F**). Arrow indicates that Nogo-B expression was lower in the smooth muscle layer in chronic asthmatic mice than that in control mice. Original magnification: ×100 for the large pictures and ×400 for the smaller inserts located in the right bottom of the large ones. **G**, mRNA expression of Nogo-B in the lung of control and chronic asthmatic mouse model. **H**, Western blot analysis demonstrated expression of Nogo-B in the lung of controls and chronic asthmatic mice. **I**, Protein intensity detected by Western blot with Nogo-B antibody and GAPDH. **J, **Effects of PDGF on Nogo-B expression in vitro. Data are mean ± SD, n = 7, *P < 0.05 as indicated.

To investigate the role of Nogo-B in airway remodeling in asthma, we constructed a mouse model of chronic asthma. Evident airway inflammation and airway thickening could be observed in mice with chronic asthma (Figure 1A-B). The asthmatic mice also had significantly increased expression of SM-22 (Figure 1C-D), a specific marker of differentiated ASM cells in the airway, indicating evidence of airway smooth muscle remodeling. Immunohistochemistry revealed that Nogo-B was widely expressed in the lung, especially abundant in epithelium, alveolar epithelial cells, and airway smooth muscle cells. In chronic asthmatic mice, the distribution of Nogo-B was not altered. However, there was a significant decrease in the airway smooth muscle layer (Figure 1E-F). Additionally, Realtime analysis revealed a significant reduction of lung Nogo-B mRNA expression in chronic asthmatic mice (Figure 1G), in accordance with this, Western blotting analysis of the total proteins collected from the lung homogenates showed that Nogo-B expression was approximately 3.08-fold lower in chronic asthmatic mice than in control mice (Figure 1H-I), indicating that Nogo-B may play a role in airway smooth muscle remodeling in asthma. However, incubation of cultured HBSMCs with an increasing concentration of PDGF-BB for up to 48 h resulted no obvious change of Nogo-B as evidenced by western blotting analysis (Figure 1J).

### RNAi for Nogo-B expression

**Figure 2:**
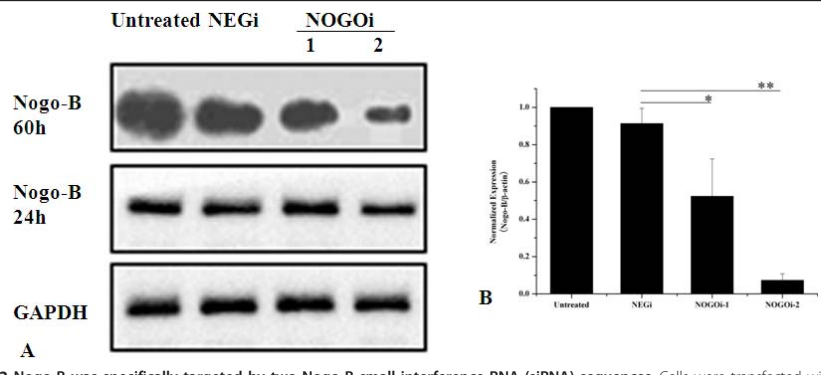
**Nogo-B was specifically targeted by two Nogo-B small interference RNA (siRNA) sequences**. Cells were transfected with 10 nM Nogo-B siRNA. Following transfection, cells were lysed for Western blot analysis at 24 and 60 h (**A**) or for RNA isolation at 60 h and real-time PCR analysis (**B**). n = 3, *P < 0.05, **P < 0.01 compared with NEGi.

To determine the role of Nogo-B in airway smooth muscle cells, we used a siRNA approach to knockdown Nogo-B expression in HBSMCs *in vitr*o. Transfection of cells with two different Nogo-B siRNA (NOGOi) sequences resulted in knock-down of Nogo-B protein expression, as determined by Western blotting analysis (Figure 2A). Transfection of negative control siRNA (NEGi) had no effect on Nogo-B expression levels. Additionally, NOGOi-transfected cells showed a 96% reduction in Nogo-B mRNA compared to NEGi-transfected cells 60 h post-transfection, as determined by quantitative real-time PCR (Figure 2B).

### Effects of Nogo-B on proliferation and migration of HBSMCs

**Figure 3:**
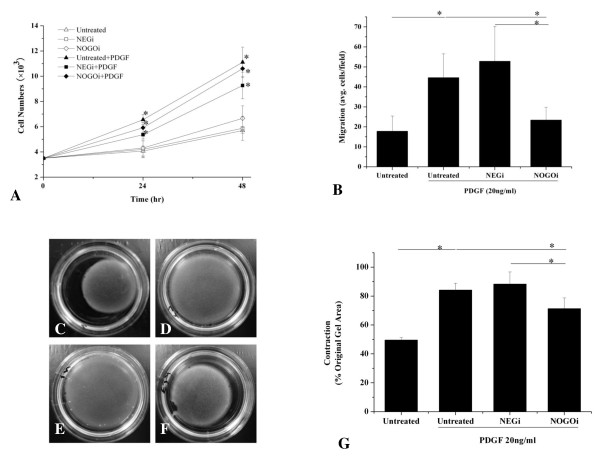
**Effects of Nogo-B knock-down on proliferation, migration and contraction of HBSMCs**. **A**, Untransfected cells or HBSMCs transfected with NEGi or NOGOi-2 for 48 h were starved overnight to achieve growth arrestment and treated with PDGF-BB (20 ng/ml). Cell numbers were counted by proliferation assay. *P < 0.05 compared with untreated control. **B**, Untransfected cells or HBSMCs transfected with NEGi or NOGOi-2 for 48 hours were starved overnight a treated with PDGF-BB (20 ng/ml) in SmGM to induce migration. **C-F**, representative pictures of contraction of untreated HBSMCs (C) or PDGF-treated untransfected control (**D**), NEGi transfected (**E**), and NOGOi-2 transfected (**F**) HBSMCs. **G**, Statistic analysis of the effects of Nogo-B inhibition on the contraction of HBSMsC. All data are mean ± SD, n = 3 for triplicate experiments, *P < 0.05, **P < 0.01 as indicated.

In the next step, we examined the effects of Nogo-B on PDGF-induced abnormalities of HBSMCs *in vitro*. HBSMCs, pretreated with either NEGi or NOGOi-2 for 48 h, were starved overnight, reseeded onto a 96-well plate at a density of 3.5 × 10^3 ^in 2% FBS SmGM and incubated with PDGF-BB (20 ng/ml). Stimulation of HBSMCs with PDGF for 24 h and 48 h resulted in significant increase in cell numbers, both in untransfected and transfected cells, but there was no significant difference between the NEGi and the NOGOi-2 treated groups. These results demonstrated that down-regulation of Nogo-B had no significant effect on the proliferation of HBSMCs at either time point (Figure 3A). Next, we characterized the effects of Nogo-B on PDGF-induced HBSMC migration. As shown in Figure 3B, PDGF (20 ng/ml) resulted in an approximately 4.4-fold increase in migration of HBSMCs. Also, cells pretreated with NEGi for 60 h showed a marked increase in migration after PDGF induction, similar to the untreated controls. Knockdown of Nogo-B significantly inhibited the migration of HBSMCs, as much as 2.3-fold compared to the NEGi group. These findings suggest that Nogo-B is necessary for the migration of HBSMCs.

### Effects of Nogo-B on the contraction of HBSMCs

It is believed that PDGF can switch SMC to an undifferentiated phenotype that exhibits diminished contractility. Therefore, using a gel contraction assay, we tested the role of Nogo-B on the contraction of HBSMCs pretreated with PDGF. Cells pretreated with PDGF (20 ng/ml) exhibited reduced contractility in NEGi controls and the untreated controls, as identified from gel surface area. In the NOGOi-2 group, however, the gel surface was much smaller than in the NEGi controls and untreated controls, indicating an increased contractility after Nogo-B down-regulation (Figure 3C-G).

### Proteomic analysis revealed changes in MYL-9 and ARPC2/3 after Nogo-B knock-down

**Figure 4:**
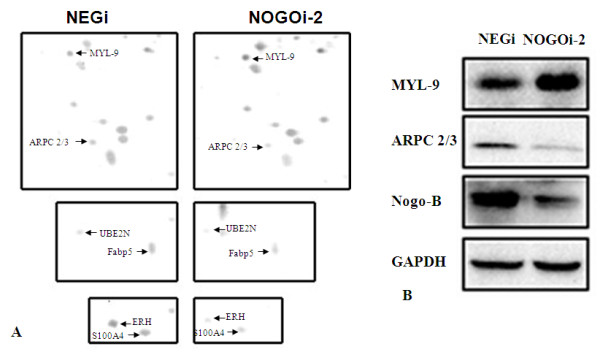
**Identification and verification of changes in proteins expression after Nogo-B knockdown by proteomic assay**. **A**, Enlargements of silver-stained gels. Representative areas of 2-dimensional gel from NEGi and NOGOi-2 lysates highlighting the quantitative differences in the images. **B**, expression of ARPC 2/3 and MYL-9 proteins as detected by Western blot after Nogo-B knockdown (in agreement with the proteomic findings). This verification experiment was repeated for three times.

To more clearly define the role of Nogo-B on the modulation of PDGF-induced SMC migration and contraction, we performed a proteomic analysis. Two-dimensional electrophoresis was performed and approximately 1,000 spots, on average, were detected for NEGi or NOGOi-2 treated HBSMCs in silver-stained gels using ImageMaster. The proteins in the high molecular weight region of the 2D gels could not be separated clearly. In a low molecular weight region, a mean of 350 spots were matched. In comparison with the control group, 15 spots in the NOGOi-2 HBSMC group demonstrated a relative concentration changed of more than 3-fold. Enlarged silver-stained gels highlight the quantitative differences in the images (Figure 4A), here, only the successfully identified spots are shown). Numbered spots were excised and subjected to in-gel digestion. Protein identifications, as obtained by MALDI-TOF MS, are listed in Table 1. We focused our interests on two of the six proteins successfully identified, including myosin regulatory light chain 9 isoform a (MYL-9) and actin related protein 2/3 complex subunit 5 (ARPC 2/3), which, are the key proteins in the processed of SMC contraction and migration. To further validate the proteomic data, we again performed RNAi in the HBSMCs and analyzed the protein expression by Western blotting. In accordance with the results found in the proteomic analysis, Figure 4B demonstrates that the expression of ARPC 2/3 decreased, while MYL-9 expression increased after Nogo-B knock-down. These results convinced us that the expression of ARPC 2/3 and MYL-9 changes after Nogo-B knock-down.

**Table 1:** Identification of the differentially expressed protein after Nogo-B knock-down

No	Protein	Mr	PI	Accession No of NCBI	Fold Change	Sequence Coverage/Score
1	fatty acid binding protein 5	15155	6.6	gi|4557581	3.32 ↓	31%/199
2	ubiquitin-conjugating enzyme E2N	17127	6.13	gi|4507793	4.22 ↓	82%/342
3	myosin regulatory light chain 9 isoform a	19814	4.8	gi|29568111	8.05 ↑	51%/251
4	actin related protein 2/3 complex subunit 5	16310	5.47	gi|5031593	3.32 ↓	54%/259
5	enhancer of rudimentary homolog	12251	5.63	gi|4758302	14.3 ↓	30%/211
6	S100 calcium-binding protein A4	11721	5.85	gi|4506765	5.22 ↓	58%/276

(↓ stands for down-regulation; ↑ stands for up-regulation.)

## Discussion

In the present study, we showed that Nogo-B was down-regulated in the smooth muscle layers of the airways of chronic asthmatic mice. In addition, the endogenous expression of Nogo-B was necessary for airway smooth muscle cell migration and contraction, but had limited effect on proliferation of the cells. Furthermore, we revealed for the first time that ARPC 2/3 and MYL-9 may be two of the factors responsible for the functional effects of Nogo-B on airway smooth muscle cells. Our results suggest that Nogo-B plays an important role in regulating airway smooth muscle cells and, therefore, participates in airway remodeling in asthma.

We demonstrated that Nogo-B was significantly down-regulated in the lungs of chronic asthmatic mice. Also, immunohistochemistry indicated that expression of Nogo-B decreased in the airways of smooth muscle layer of chronic asthmatic mice. These results strongly implicate Nogo-B in asthmatic airway smooth muscle remodeling. Nogo-B is a 37 kDa protein belonging to the RTN4 family. The importance of Nogo-A as a potent inhibitor was initially described during axonal growth in the central nervous system [9,11,16,17]. Nogo-B, which shares homology with Nogo-A, was then identified outside the central nervous system [10]. Previous studies have shown that down-regulation of Nogo-B most likely occurs under conditions of trauma and inflammation and, therefore, is responsible for multiple pathological conditions such as atherosclerosis, aortic aneurysms formation, and vascular regeneration after vessel injury [11,18-21]. However, up-regulation of Nogo-B has also been reported in inflammation initiated by ischemia and is necessary for wound healing [12]. These studies suggest that Nogo-B may play a complex role in different stages and types of inflammation. In the case of airway remodeling of asthma, decreased Nogo-B may also result from inflammation and a repair response. A similar phenomenon was also observed in both a mouse model of acute asthma and in severe asthmatic patients [22]. In the next step, we are going to construct the chronic asthma models of mice on Nogo-B deficient mice and hope to find out the exact role of Nogo-B on airway smooth muscle remodeling.

Nogo-B was originally identified as an apoptosis-inducing protein through multiple pathways [23,24] and then was know as a regulator of vascular remodeling [12]. As both proliferation and apoptosis are believed to contribute to airway smooth muscle remodeling in asthma [25], we tested whether Nogo-B played a role in airway remodeling. We found that down-regulation of Nogo-B had no effects on the proliferation of HBSMCs. Our findings confirm the result of a previous investigation demonstrating that stable transfectants overexpressing Nogo-B did not differ significantly from the respective parental wild-type of control cell lines both in respect to cell proliferation and to spontaneous apoptosis induced by staurosporine and tunicamycin [26]. These results suggest that Nogo-B may not exert its role through modulation on proliferation and apoptosis.

Increased emigration of airway smooth muscle cells was also thought to participate in airway remodeling in asthma [27]. We showed that down-regulation of Nogo-B significantly inhibited PDGF-induced migration of HBSMCs, underscoring a role for Nogo-B in airway smooth muscle remodeling. Previous studies demonstrated that Nogo-B played a complex role in cell migration. For example, Nogo-B N-terminal peptides promote migration of endothelial cells while inhibiting migration of vascular muscle cells [11], and Nogo-B deficient macrophages exhibited deficiency in migration and spreading [12]. Three mechanisms, besides different cell lines, may account for such differences. Firstly, genomic studies have revealed that Nogo-B deficient mice show significantly decreased expression of Nogo-B receptors, which are vital for chemotaxis and morphogenesis of endothelial cells [12]. Secondly, PDGF receptors are down-regulated after Nogo-B knock-down, which definitely attenuates the effects of PDGF-induced migration [12]. Finally, we report for the first time that down-regulation of Nogo-B inhibites the expression of ARPC 2/3 subunit 5. ARPC 2/3 subunit 5 is a family member of actin related protein complex 2/3 and plays an important role in actin-filament nucleation, and ARPC 2/3 inhibition results in diminished migration [28,29]. Taken together, these mechanisms also explain the inhibitory effect on migration after Nogo-B knock-down in our experiment.

Interestingly, we demonstrated for the first time that Nogo-B knock-down may increased the contraction of HBSMCs by up-regulating MYL-9. MYL-9, also know as myosin light chain 2 (MLC-2), is a 20 kDa protein that can be phosphorylated by myosin light chain kinase in the presence of calcium and calmodulin and increases the actin-activated ATPase activities of myosins [30]. Phosphorylation of MYL-9 initiates the contraction of smooth muscle cells [31]. When it is up-regulated, more contract-related proteins are recruited and the capability and sensitivity of contraction is greatly enhanced. Our results from proteomic analysis provide an exciting possible explanation of how Nogo-B modulating migration and contraction. However, the precise mechanisms deserve further investigation.

## Conclusions

In conclusion, the present study implicates Nogo-B in airway remodeling in asthma. Endogenous Nogo-B, which may exert its effects through ARPC 2/3 and MYL-9, is necessary for the migration and contraction of airway smooth muscle cells. Further studies are needed to clarify the therapeutic potential of Nogo-B during airway remodeling in asthma.

## Abbreviations

ASM: airway smooth muscle; Nogo-B: Inhibitor of neurite outgrowth-B; HBSMC: human bronchial smooth muscle cells

## Competing interests

The authors declare that they have no competing interests.

## Authors' contributions

XWJ carried out the animal and part of the cytobiological experiments and drafted the manuscript. HWJ carried out the cell culture. SY carried out the molecular biological experiments. NYY participated in the design of the study and performed the statistical analysis. CZL participated in the molecular biological experiments and helped to draft the manuscript. LQ designed the study and participated in drafting the manuscript. All authors read and approved the final manuscript.
